# Immunology of simultaneous liver and kidney transplants with identification and prevention of rejection

**DOI:** 10.3389/frtra.2022.991546

**Published:** 2022-11-01

**Authors:** Sandesh Parajuli, Luis G. Hidalgo, David Foley

**Affiliations:** ^1^Division of Nephrology, Department of Medicine, University of Wisconsin School of Medicine and Public Health, Madison, WI, United States; ^2^Division of Transplantation, Department of Surgery, University of Wisconsin School of Medicine and Public Health, Madison, WI, United States

**Keywords:** simultaneous liver and kidney transplant, immunology, rejection, sensitization, donor-specific antibodies

## Abstract

Simultaneous liver and kidney (SLK) transplantation is considered the best treatment modality among selected patients with both chronic kidney disease (CKD) and end-stage liver disease (ESLD). Since the first SLK transplant in 1983, the number of SLK transplants has increased worldwide, and particularly in the United States since the implementation of the MELD system in 2002. SLK transplants are considered a relatively low immunological risk procedure evidenced by multiple studies displaying the immunomodulatory properties of the liver on the immune system of SLK recipients. SLK recipients demonstrate lower rates of both cellular and antibody-mediated rejection on the kidney allograft when compared to kidney transplant-alone recipients. Therefore, SLK transplants in the setting of preformed donor-specific HLA antibodies (DSA) are a common practice, at many centers. Acceptance and transplantation of SLKs are based solely on ABO compatibility without much consideration of crossmatch results or DSA levels. However, some studies suggest an increased risk for rejection for SLK recipients transplanted across high levels of pre-formed HLA DSA. Despite this, there is no consensus regarding acceptable levels of pre-formed DSA, the role of pre-transplant desensitization, splenectomy, or immunosuppressive management in this unique population. Also, the impact of post-transplant DSA monitoring on long-term outcomes is not well-studied in SLK recipients. In this article, we review recent and relevant past articles in this field with a focus on the immunological risk factors among SLK recipients, and strategies to mitigate the negative outcomes among them.

## Introduction

Simultaneous liver and kidney (SLK) transplantation is considered the best treatment modality among selected patients with both chronic kidney disease (CKD) and end-stage liver disease (ESLD) (1). Kidney dysfunction among liver transplant candidates and recipients has deleterious effects on both patient and graft survival (2, 3). As serum creatinine is a prominent variable in the Model For End-Stage Liver Disease (MELD) equation, patients receiving liver offers have had an increasing burden of renal dysfunction since the adoption of the MELD-based allocation policy in 2002 (1). As a result, the frequency of SLK transplantation compared to liver transplantation alone (LTA) has also increased in the current era. In 2016, 9.3% of all liver transplants were performed in the form of SLK compared to 2.7% in 2000 (1).

There is substantial clinical evidence displaying the immunoprotective effects of the liver allograft on the kidney allograft among SLK transplant recipients compared to the kidney transplant alone (4, 5). Seeing that levels of preformed donor-specific antibody (DSA) to human leukocyte antigen (HLA) often decrease or disappear following SLK transplant, this protective effect is thought to be due to hepatic absorption of circulating anti-HLA antibody (5, 6). Although not well-studied, SLK transplants in the setting of preformed anti-HLA DSA is a common practice (7). In this review, we summarize the management challenges of immunological risk among SLK recipients focusing on the pre-transplant, peri-transplant, and post-transplant periods with particular emphasis on short and long-term outcomes.

## History of simultaneous liver and kidney transplant

The first-ever SLK transplant was performed on December 28, 1983, in a 32-year-old man who was also a previous kidney transplant recipient and had failed kidney allograft along with ESLD due to hepatitis B virus (HBV) infection in Austria by Margreiter et al. (8, 9). Within the next 17 years, i.e., by the end of 2000, 22 SLK were transplanted in the same center with excellent outcomes. Their first five patients were still alive after 167–205 months post-transplant (8). Even from early experimental work it was known that liver allografts have the potential to induce immune tolerance, at least in a porcine model (10). Even from the earlier series of SLK transplants Margreiter et al. reported one patient with 100% preformed HLA antibodies and positive lymphocytotoxic crossmatch never developed kidney allograft rejection (8). However, some SLK recipients were not able to demonstrate the protective effect of the liver transplant. In 1996, Katznelson et al. reported 248 SLK recipients compared with 206 kidney-only transplant recipients from the same donor and found similar death censored graft survival at 81 and 78% between the two groups. The authors concluded that the liver neither protects the kidney from rejection nor improves kidney allograft function among SLK recipients (11).

However, most of the recent studies support that among SLK recipients, the liver graft is a key predictive factor of lower cellular and antibody-mediated rejection and renal function decline of the kidney allograft (12–14). SLK recipients have lower circulating effector memory T cells, proliferative responses to donor cells, and frequency of interferon-γ-producing alloreactive T cells compared with kidney-alone recipients (15). This is also supported by the fact that a kidney transplant from a different donor after a liver transplant results in a higher rate of kidney rejection compared to kidney rejection among SLK recipients, supporting the importance of biological similarity (16).

## Liver transplantation immune tolerance mechanisms

**Figure 1:**
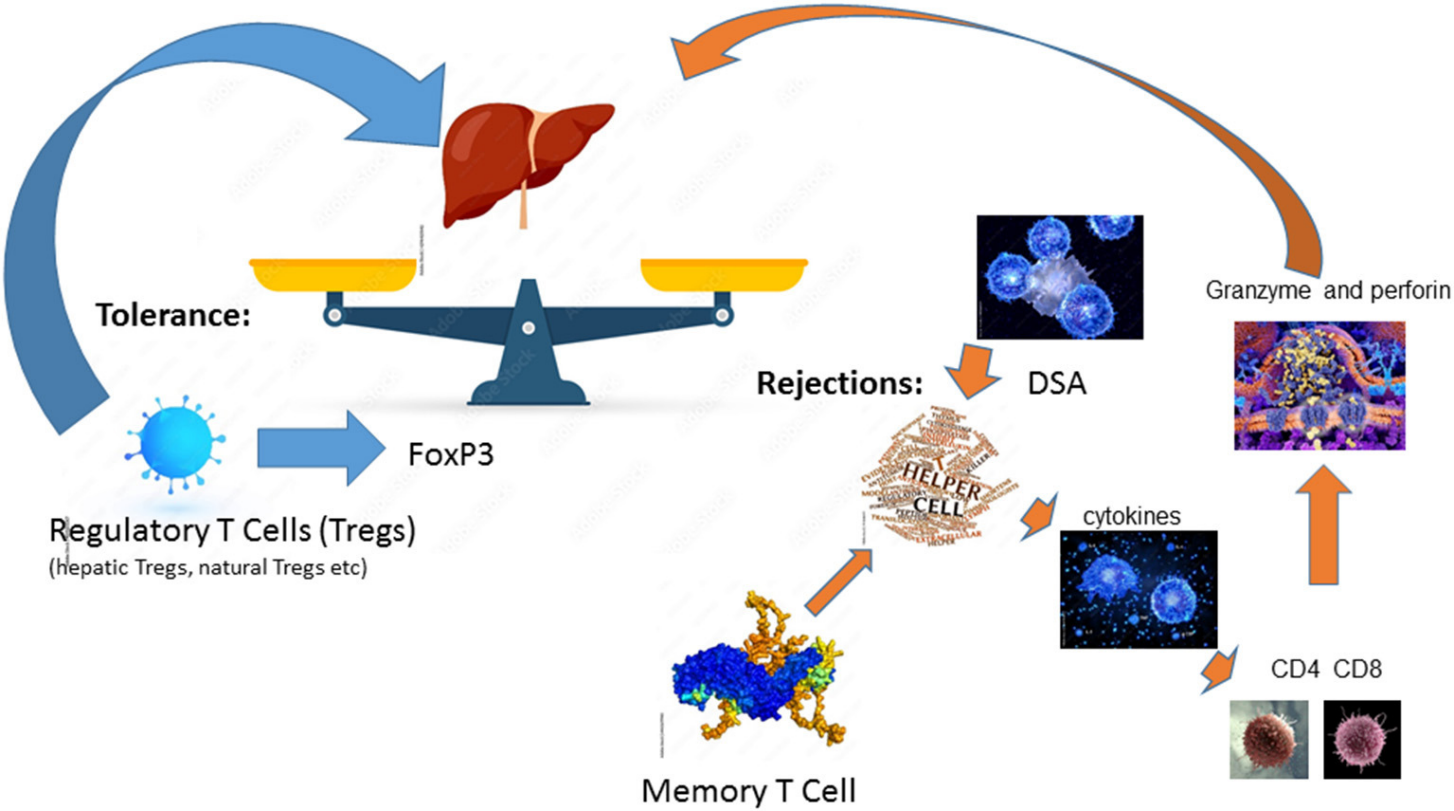
Fine balance between rejection and tolerance.

Among solid organ transplantation, the liver allograft is considered to be the most tolerogenic immunoregulatory organ (17). Among liver-only transplant recipients, it has been reported that ~20% of stable and carefully selected recipients, can be weaned off all immunosuppressive medication (18). After a liver transplant, the alloantigen, mainly the HLA is pervasive, persists for life, and can be presented by antigen-presenting cells (APCs) at numerous sites (17). Thus, liver allograft rejection is mainly caused by the mismatch of HLAs even among liver transplant recipients and requires lifelong immunosuppression. Alloantigen-activated helper T cells secrete cytokines including TNFα, IFNγ, and IL-2 to further enhance the innate immune responses upon the alloantigen challenge. They also stimulate effector CD4 T cells and cytotoxic CD8 T cells to express granzyme and perforin, thereby attacking the liver allograft (17). In addition to the cell-mediated acute rejection, DSA-mediated humoral immune response can lead to acute rejection and chronic rejections. DSA-mediated rejection is initiated by and in conjunction with T cell-mediated alloimmunity (19, 20). Memory T cells, especially donor-antigen specific memory T cells, are a major obstacle to successful tolerance induction (17). In reverse, of conventional T cells, regulatory T cells (Tregs), which are a specialized CD4 T cell subpopulation with the key transcription factor FoxP3 expression, are found to play an essential role in operational tolerance post solid organ transplantation (17). There are several different Tregs including natural Tregs (nTregs), induced Tregs (iTregs), IL10 producing Type 1 regulatory T cells (Tr1 cells), and TGF-β producing Th3 cells (17). Hepatic iTregs are the major source of peripheral Tregs and lead to transplant tolerance together with nTregs in both humans and mice (17). Tolerance is a fine balance between two opposing forces of the host immune system and liver-mediated immune regulation (21). This balance can be tilted toward rejection by the stimulation of the immune system due to tissue damage or after exposure to endotoxins from infectious agents or Toll-Like Receptors resulting from ischemia-reperfusion injury, thereby upregulation of class II HLA on hepatic endothelial cells (22, 23). This fine balance of alloreactive and tolerance is summarized in Figure 1.

Among SLK recipients, there is a distinct expression of genes for T cell recruiting cytokines among tolerant recipients, with a large number of CD3+ T cells and macrophages in the liver allograft but only a few in the simultaneously implanted kidney allograft (24). Increased expression of chemokines in the liver attracts alloreactive T cells that are subsequently destroyed by coming in contact with various liver cells inherently programmed toward tolerance induction (21). With this, SLK recipients have a lower frequency of circulating CD8+, activated CD4+, and effector memory T cells, compared to the kidney transplant alone recipient (14). These mechanisms underlying the liver's protective role do not only operate inside the liver but extend to the kidney among SLK recipients, as there are distinct gene expressions seen in the real-time reverse transcriptase-polymerase (12).

Liver-induced protection against the immune system is multifactorial and includes: microchimerism, deficient antigen presentation due to lack of costimulation, expression of inhibitory molecules, deletion of activated recipient T cells in the liver, the deposition of a large antigen load, active secretion of HLA molecules neutralizing alloantibody, and generation of Tregs in peripheral lymph nodes (21).

## Recipients with pre-transplant HLA DSA and outcomes

SLK transplants benefit from the same advantages as liver transplants alone based on the liver's unique ability to withstand damage from certain types of HLA antibodies. The liver displays at least two types of immunoregulatory properties. One property allows for the rapid absorption and removal of DSA from the recipient's blood and is likely due to high HLA expression within compartments in the liver. The other property is more properly immunoregulatory and actively decreases the degree of donor-specific humoral and cellular responses through incompletely understood mechanisms (7).

A factor that impacts the degree of immunologic risk is the presence of pre-existing DSA in the recipient. Hibi et al., show that recipient sensitization in SLK transplantation, defined as the presence of Panel Reactive Antibody (PRA) >20%, was a predisposing factor affecting kidney graft survival (25). It is important to note that the degree of HLA antibody information in that study was limited and neither DSA nor donor-specific crossmatch information was noted. The absence of this information was surprising seeing that immunological risk was attributed to a previous transplant which greatly increases the risk for post-transplant sensitization. The likelihood of encountering pre-existing DSA increases in proportion to the degree of recipient sensitization.

Kidney transplant studies, where histocompatibility is more closely monitored, demonstrate that highly sensitized patients based on PRA levels may be transplanted without an increased risk when DSA is absent. A great example of how PRA is an incomplete metric for immunologic risk is the success of the Eurotransplant Acceptable Mismatch program where highly sensitized patients are transplanted with compatible donors in the absence of DSA despite their sensitization status and display similar long-term graft survival rates to those of unsensitized patients (26). The historic focus on the PRA status of a SLK and liver transplant recipient may stem from the inaccuracies previously observed with early HLA antibody detection assays and HLA typing assays with limited resolution. With the advent of Luminex single-antigen bead technology and greatly improved molecular HLA typing methods, DSA is currently easier to identify and characterize than before and is considered the gold standard for HLA antibody identification. This will be realized in SLK transplantation as more centers begin to adopt SLK histocompatibility practices to match those of kidney transplants alone. However, centers may not want to put limitations on their potential SLK recipients by employing the HLA restrictions used for kidney transplants alone.

Kidney transplants performed across pre-existing DSA display higher rates of early and late antibody-mediated rejection (ABMR), and lower graft survival. In contrast, kidney allografts transplanted simultaneously with a liver in the setting of pre-existing DSA generally do not sustain antibody-mediated damage from even high levels of pre-existing DSA (7, 27). The main misconception of performing SLK or liver transplants alone across pre-existing DSA is that the liver can modulate any pre-existing DSA. However, studies showing detailed HLA antibody measurements clearly demonstrate that the liver is best able to absorb class I HLA antibodies, but is less effective with class II HLA antibodies (6, 7, 28, 29).

The inability of liver transplants to absorb class II DSA has clinical implications for both kidney and liver allografts in SLK transplants. Indeed, multiple studies demonstrate that when class II DSA levels remain high post-transplant, ABMR in the kidney is likely to ensue (30). Even in cases where patients with pre-existing, high-level class II DSA receive different induction therapy suitable for high immunologic risk patients, the risk for kidney ABMR is not avoided (30). Other studies have demonstrated increased rates of rejection and higher mortality rates in SLK recipients but no specific association with class I vs. class II DSA (28), which suggests that further diagnostic refinement remains. Although liver ABMR is described and felt to be less injurious to the allograft compared to ABMR in the kidney allograft, the livers are not completely spared from antibody-mediated damage. Livers may be more resistant to ABMR potentially due to the size of the liver allograft as well as the liver's regenerative abilities. However, ABMR in the liver is more difficult to recognize and diagnose, a reflection of the unfinished evolution and acceptance of the consensus of features that define liver ABMR (31, 32).

The exact mechanism by which liver allografts remove DSA from circulation remains under investigation. Early studies suggest a role for non-parenchymal liver cells and specifically Kupffer cells with their ability to bind to DSA and complement components (33) and their broad expression of Fc receptors (34). The preferential adsorption of class I antibodies may be due to HLA expression levels in the liver where high class I expression but limited class II are observed in animal models of liver transplantation (35). The reduced HLA class II expression on liver cells preferentially targeted in rejection combined with the limited overall number of liver cells capable of class II expression may underlie the liver's limited ability to absorb class II antibodies. The limited expression of class II also explains the increased susceptibility to class II antibodies resulting in a high antibody: target ratio that maximizes antibody-mediated immune effector activity.

The current understanding of pre-existing DSA in SLK transplantation suggests that class I DSA at the time of transplant poses little risk to either transplant. When high levels of class I DSA are crossed, staggering the transplants to allow the liver time to absorb the DSA before implantation of the kidney transplant limits the degree of antibody-mediated damage to the kidney and may prevent ABMR (36). The same cannot be said for class II DSA where minimal adsorption by the liver is observed and immunomodulation leading to decreases in DSA requires months. This problem may be even worse if the class II DSA is directed against HLA-DQ targeting a repeat DQ mismatch since the levels of DQ DSA tend to be highest among all HLA loci (37).

## HLA mismatch and outcomes among SLK recipients

The literature on HLA mismatch in SLK transplantation is limited. A role for HLA mismatch is controversial with some studies demonstrating an effect on the development of *de novo* DSA and/or T cell-mediated rejection (TCMR) (38, 39) while others find no impact on rejection or outcomes (40). However, it is important to decipher the impact of HLA mismatch on the alloimmune aspects vs. the different immunologic etiologies leading to the need for a liver/SLK transplant including Hepatitis B infection, or other autoimmune disorders.

The degree of HLA mismatch in SLK transplants dictates the extent of alloimmunity generated by the recipient. DSA monitoring of SLK recipients demonstrates that *de novo* DSA develops in these patients and primarily targets HLA class II antigens, particularly HLA-DQ (41), as described for other organs. Newer algorithms for HLA matching focus on the degree of HLA class II match between donor and recipient and use a more granular approach compared to conventional antigen matches. These newer methods are referred to as “molecular HLA mismatch” and have been well-studied in kidney transplantation. The value of HLA matching is in the prevention of *de novo* DSA and subsequent development of ABMR, both parameters that are carefully assessed in kidney transplantation but less so in liver transplants. However, cellular rejection is commonly observed in liver transplantation and the risk for this type of rejection is also linked to the degree of molecular HLA mismatch (42).

Multiple tools are available to calculate molecular HLA mismatch. One common tool is called HLA Matchmaker which calculates the differences in functional epitopes or “eplets” between donor and recipient (43). Another tool is the Predicted Indirectly ReCognizable HLA Epitopes (PIRCHE-II) algorithm which examines predicted HLA-derived epitopes recognized by T cells through indirect antigen recognition (44). Recent studies demonstrate variable results using these newer HLA mismatch tools in liver transplants. In pediatric liver transplantation, the eplet load predicts the risk for *de novo* DSA (45). In a single-center study of 736 primary liver transplants, neither the PIRCHE-II or eplet scores were associated with patient mortality, graft loss, or rejection (46). When examined in liver transplant recipients on CNI-free maintenance immunosuppression, PIRCHE-II score and donor age were independent risk factors for liver graft survival in CNI-free patients (47). These varying results argue that additional studies are needed to determine the true value of modern HLA matching in liver and SLK transplantation.

## Desensitization strategies among highly sensitized SLK recipients

The data on SLK transplants in highly sensitized recipients with very high preformed DSA levels is scant and is mainly based on a few reports often lacking detailed immunocompatibility and pathology assessments. Studies comparing sensitized SLK recipients with non-sensitized SLK recipients did not find any difference in the rate of antibody-mediated rejection rates, kidney graft survival, or patient survival (5, 7). Therefore, at many transplant centers, the decision-making for donor and recipient matching in SLK transplants is based solely on ABO compatibility without consideration of crossmatch results or level of HLA DSA level (5, 48, 49).

The immunosuppression protocols and induction treatments for SLK transplants have not been well-established, and there is no clear evidence supporting any specific protocol even in highly sensitized recipients. In one study, repeat crossmatch testing on sera obtained 1 h after liver transplantation revealed conversion from positive to negative results, suggesting the liver reduces HLA DSA by absorption (48). While in another case report, the prospective DSA analysis acquired 1 month after SLK transplant demonstrated a significant decline in DSA level in one highly sensitized recipient (7). In the same case report, the authors report delaying kidney transplant by 6 h after liver transplant with the hope to allow more time for the liver allograft to absorb DSA (7). That patient also received rituximab for induction and eculizumab before reperfusion of kidney allograft. Cytotoxic and flow cross matches repeated 6 h after liver transplant and before the implantation of the kidney allograft, remained markedly positive. The patient's course was complicated with delayed graft function of the kidney and also abnormal liver function tests. Kidney biopsy on postoperative days 2 and 17 showed the features of antibody-mediated injury. HLA antibody elutions of the kidney and liver transplant biopsies were analyzed by single antigen assay and demonstrated the presence of class I and II DSA in both liver and kidney biopsies and were treated further with weekly doses of eculizumab. With all these treatments, the patient had an excellent liver and kidney allograft function at 1-year post-transplant. This case suggests that the presence of extremely high preformed class I and II DSA, among SLK transplants, may not prevent antibody-mediated rejection in the kidney allograft, even with a 6-h delay between liver and kidney transplants from the same donor. Also, DSA levels may be high enough in some patients that the liver cannot protect the kidney by DSA absorption and the use of rituximab and eculizumab may be helpful. Despite this, there is no consensus among transplant centers on the use of induction agents among SLK recipients.

Analysis of the Scientific Registry of Transplant Recipients showed that only 14–19% undergoing SLK transplants usually receive lymphocyte-depleting agents as induction even among sensitized recipients (49). Most of the centers use only an interleukin-2 receptor antagonist, such as basiliximab for induction in SLK transplants (50). The role of pre and post-transplant plasma exchange among highly sensitized SLK recipients is also not well-described. Similarly, the role of splenectomy even among liver-only transplant candidates is controversial and is not well-reported among SLK recipients (51). The spleen may have beneficial effects on long-term T lymphocyte modulation as splenectomy may reduce acute rejection and has been utilized in ABO-incompatible liver transplants to prevent antibody-mediated rejection (52–54). Also, the role of splenectomy in the prevention of rejection is not well-documented.

In one single-center study among liver transplant recipients, Golse et al. (51) compared 47 liver transplant recipients with simultaneous splenectomy with 94 liver transplant recipients without splenectomy and did not find any significant differences in the rate of rejection or hospital morbidity. Unfortunately, the splenectomy group had a longer operative time, greater blood loss, and a significantly higher incidence of *de novo* portal vein thrombosis and infection rate compared to the liver transplant group without splenectomy. From the same cohort, of 47 liver transplant recipients with splenectomy, two were recipients of SLK transplants (51). The details of the indications and outcomes among these two SLK recipients were not provided. Similarly, in another study among 40 liver transplant recipients with splenectomy, authors reported a higher rate of 1 month and 1-year patient mortality, along with sepsis compared to the liver transplant without splenectomy (55). However, some reports support splenectomy in the liver transplant recipient mainly among the living donor liver transplant recipient (56, 57). Given the limited data in published studies, splenectomy is not commonly performed or recommended among SLK recipients to mitigate the risks of rejection in patients with high DSA. Induction with rituximab and eculizumab, along with eculizumab maintenance therapy could be another option among highly sensitized SLK recipients (7), with less morbidity compared to the splenectomy.

## Induction and maintenance of immunosuppression

There are wide variations in the use of induction and maintenance immunosuppression among SLK recipients. In one study, analyzing the Organ Procurement and Transplant Network registry data from 1996 to 2016, Kamal et al. (58) reported, that of 5,172 SLK recipients, 941 (18%) received T-cell depletion induction, 1,635 (32%) received interleukin 2 receptor antagonist, and 2,596 (50%) received no induction. From the same study, it seems recipients highly sensitized as indicated by higher PRA were likely to receive T-cell depletion induction with a mean PRA of 6.2 ± 21 vs. 5.1 ± 17.5 in the interleukin 2 receptor antagonist group vs. 3.9 ± 16.4 in no induction group (*p* = 0.006) (58). However, the rates of either kidney or liver rejections at 1 year were similar among the three induction groups.

Similarly, there is extensive center-level variation regarding long-term immunosuppression maintenance therapy (59). The hallmark maintenance immunosuppressive agents include calcineurin inhibitor with tacrolimus, antimetabolite, and plus/minus steroids (60). A recent study among 4,184 SLK recipients found that the implementation of steroid-sparing regimens increased in incidence from 16.1% at discharge to 88.0% 5 years post-transplant (60). From the same study, authors suggested, that a steroid-sparing regimen appears to be safe and effective. However, in another study, recipients of T-cell induction and CNI maintenance were associated with decreased patient, liver, and kidney allograft survival (58). Although there are still many knowledge gaps to address induction and maintenance of immunosuppression among highly sensitized SLK recipients, however, other factors and effects of comorbidities including hepatitis C virus status, dialysis time, prior transplants as sensitizing events, current immunosuppressive therapy, and the status and quality of the liver allograft should be considered and managed case by case (59).

We recommend SLK recipients with pre-transplant DSA against class I antigen only could be considered a lower immunological risk and could proceed with SLK transplant without induction and triple immunosuppression maintenance with CNI, antimetabolites, and steroids. If no rejection of either graft by 1-year post-transplant could consider stopping steroids by 1-year post-transplant. Among recipients with pre-transplant DSA against class II antigen, should be considered high immunological risk for rejections, and should receive either a T cell depleting agent for induction immunosuppression if the patient can tolerate it. or an interleukin 2 receptor antagonist, followed by long-term triple immunosuppression. All patients should have frequent post-transplant DSA monitoring and could be considered for steroid withdrawal after 1 year on a case-by-case basis after evaluating the history of infections, rejections, and either the disappearance or persistence of DSA.

## Kidney function after SLK transplants

To the best of our knowledge, there have been no published studies directly comparing the post-transplant kidney function in sensitized and non-sensitized SLK recipients. In one single-center study among 74 SLK recipients, Hibi et al. reported recipients with PRA > 20% to have more than 2 times higher risk of death (HR 2.8, 95% CI 1.1–7.2, *p* = 0.028) in a multivariable analysis (25). Similarly, from the same study, presence of HCV+, and PRA >20% were predictive of kidney delayed graft function (DGF) (25). Similar to kidney-only transplant recipients, the negative impact DGF among SLK recipients has been reported by Weeks et al. (61). However, another small single-center study did not find any impact on patient or allograft survival among SLK recipients with DGF (62). Similar to the kidney-only transplant recipients, the risk of kidney DGF were similar among SLK recipients with most common risk being use of donation after circulatory death organs, national sharing of organs in reference to the local, pre-transplant dialysis need and duration, and higher donor body mass index (61).

Among kidney only transplant recipients, DGF is a well-known risk factor for acute rejection. A large meta-analysis of 34 studies concluded that kidney transplant recipients with DGF had a 49% pooled incidence of acute rejection compared to 35% among patients without DGF (63). However, the effects of kidney DGF and rate of rejections of either kidney or liver allograft are not studied among SLK recipients. Sharma et al. (64) looked at the risk of CKD stage IV and higher among 570 SLK recipients at 1-, 3-, and 4-years post-transplant and reported the presence of kidney DGF as a strongly associated factor for advanced CKD (HR, 1.72; 95% CI, 1.10–2.71).

## Kidney rejection among SLK recipients

In one retrospective study among 36 SLK recipients, compared to 1,283 kidney only recipients, Hanish et al. (4) noted a significantly higher rate of rejection-free survival among SLK recipients either for cellular rejection (93%) or ABMR (96%) at 3 years post-transplant, compared to kidney only transplant recipients at 72% for TCMR and 78% for ABMR. Sensitized SLK recipients had statistically similar rejection-free survival at 3 years (75%) when compared with non-sensitized kidney-only recipients (67%, *p* = 0.55) (4). Similar findings were reported by Taner et al. (13) and concluded that SLK transplant was associated with reduced risk of alloimmune injury to the kidney allograft.

In another descriptive study among 27 SLK recipients, where eight recipients developed an acute rejection of kidney allograft, Shah et al. (30) noted that those with DSA to class II HLA with mean fluorescence intensity >10,000 were at increased risk of kidney ABMR. From the same study, two SLK recipients who had predominantly class I DSAs led to ABMR, suggesting that class I DSAs may not be as innocuous as previously considered. From the same cohort, six recipients also had liver allograft rejection, and four of these same patients did not have kidney rejection. Given all these studies, kidney rejection among SLK recipients can occur, especially among highly sensitized recipients.

## Development of *de novo* DSA and outcomes

In the field of kidney transplantation, multiple studies show the negative effect of *de novo* DSA (dnDSA) on graft survival. In kidney transplant recipients, dnDSA is considered both a marker and a contributor to an ongoing immune response, as evidenced by an increased rate of kidney function decline even before the detection of dnDSA, followed by an accelerated decline after the detection of dnDSA (65). In liver transplants alone dnDSA is considered to be an independent risk factor for patient death and liver allograft failure (66). Similar findings were found in heart transplant recipients, islet cell transplant recipients, and pancreas transplant recipients (67–69).

While the majority of SLK transplant recipients with pre-transplant DSA lose detectable class I DSA after transplant, about 10–20% develop dnDSA, mainly against class II antigen (70, 71). Class II DSA, either pre-transplant or dnDSA is independently associated with an increased risk of patient death and liver allograft failure (71). In one retrospective study among 85 SLK recipients, longer post-transplant length of stay was significantly associated with an increased risk for the development of dnDSA (72). In one study, from our institution, among 83 SLK recipients, where post-transplant DSA monitoring is protocolized, 23 patients developed dnDSA mainly against Class II HLA antigen in 22 recipients within a mean of 34.0 ± 41.3 months post-transplant (41). Of these, 23 recipients, 15 underwent a kidney biopsy within 45 days of the detection of dnDSA, including nine with stable kidney function. Of these nine protocol kidney biopsies due to dnDSA, six patients had a subclinical rejection. All these findings suggest SLK transplants may not be immune quiescent, as the risk of the development of dnDSA and kidney rejection is not uncommon. Given this post-transplant DSA monitoring would be beneficial even among SLK recipients.

## Role of donor-derived cell-free DNA among SLK recipients

Donor-derived cell-free DNA (dd-cfDNA) has been evaluated as a rejection marker in organ transplantation, mainly in kidney transplant recipients (73, 74). Although recently, dd-cfDNA as a biomarker of early rejection is getting more attention in various other solid organ recipients including the liver (75). However, to our best knowledge, the utility of dd-cfDNA among SLK recipients has not been verified in clinical practice yet.

## Summary

In summary, although SLK transplants are considered more immunotolerant compared to other solid organ transplants, the development of circulating dnDSA and resultant rejection is not uncommon in this unique population. Data from the literature suggest that performing SLK transplants in the setting of high class I DSA is not prohibitive as the liver is able to clear these antibodies from the circulation and thus mitigates the risk of antibody mediated injury to the kidney. However, high levels of class II DSA that are unable to be cleared by the liver are more concerning for the development of rejection after transplant. The literature is sparse in reproducible data supporting a consistent management strategy for preformed DSA and the development of dnDSA after SLK transplant (Table 1). As the numbers of SLK transplants continue to increase in the recent era, close monitoring of DSA and optimization of immunosuppressive medications are critical for successful long-term graft and patient survival. More studies in this field including monitoring of dd-cfDNA, predicting patients at risk for rejection, and optimization of immunosuppressive medications are needed.

**Table 1:** Some published papers and summary.

**References**	**Year of publication**	**Title**	**Conclusion**
Olausson et al. (27)	2007	Successful combined partial auxiliary liver and kidney transplantation in highly sensitized cross-match positive recipients	A simultaneous transplantation of a partial auxiliary liver graft from the same donor, with the sole purpose of protecting the kidney from harmful lymphocytotoxic antibodies, can be performed successfully despite a positive cross-match.
Ingelsten et al. (24)	2011	Postischemic inflammatory response in an auxiliary liver graft predicts renal graft outcome in sensitized patients	The protective role of the liver was associated with a proinflammatory reaction within the organ after ischemia-reperfusion. There were – an increased expression of leukocyte-recruiting chemokines in patients without rejection – Second, gene expression profiling of transplant biopsies shortly after reperfusion predicted the risk of early rejection.
Musta et al. (19)	2012	The significance of donor-specific HLA antibodies in rejection and ductopenia development in ABO compatible liver transplantation	In ABO-compatible liver transplantation humoral alloreactivity mediated by antibodies against donor HLA molecules appears to be frequently intertwined with cellular mechanisms of rejection.
Del Bello et al. (28)	2020	Combined liver-kidney transplantation with preformed anti-human leukocyte antigen donor-specific antibodies	SLK with preformed DSA is associated with lower patients' survival despite good recipients', liver, and kidney grafts outcomes.
Goggins et al. (36)	2021	Combined liver-kidney transplantation with positive crossmatch: Role of delayed kidney transplantation	In sensitized SLK transplantation recipients, the “delayed” kidney transplant approach is associated with a significant reduction in total and class I donor-specific antibodies after liver transplant before kidney transplant.
Parajuli et al. (41)	2021	The utility of donor-specific antibody monitoring and the role of kidney biopsy in simultaneous liver and kidney recipients with *de novo* donor-specific antibodies	There is a potential utility of DSA monitoring post-transplant and protocol kidney biopsy for the detection of *denovo* DSA.

## Author contributions

SP: concept, literature review, and manuscript preparation. LH: manuscript preparation and editing. DF: concept and editing. All authors contributed to the article and approved the submitted version.

## Conflict of interest

The authors declare that the research was conducted in the absence of any commercial or financial relationships that could be construed as a potential conflict of interest.

## Publisher's note

All claims expressed in this article are solely those of the authors and do not necessarily represent those of their affiliated organizations, or those of the publisher, the editors and the reviewers. Any product that may be evaluated in this article, or claim that may be made by its manufacturer, is not guaranteed or endorsed by the publisher.

## Abbreviations

ABMR: antibody-mediated rejection
APC: antigen-presenting cells
CKD: chronic kidney disease
dd-cfDNA: donor-derived cell-free DNA
DSA: donor-specific antibody
dnDSA: *de novo* DSA
ESLD: end-stage liver disease
HBV: hepatitis B virus
HLA: human leukocyte antigen
LTA: liver transplantation alone
MELD: model for end-stage liver disease
PRA: panel reactive antibody
SLK: simultaneous liver and kidney
TCMR: T cell-mediated rejection
Tregs: regulatory T cells.
